# Prehospital factors associated with out-of-hospital cardiac arrest outcomes in a metropolitan city: a 4-year multicenter study

**DOI:** 10.1186/s12873-023-00899-3

**Published:** 2023-10-26

**Authors:** Jae Yun Ahn, Hyun Wook Ryoo, Sungbae Moon, Haewon Jung, Jungbae Park, Won Kee Lee, Jong-yeon Kim, Dong Eun Lee, Jung Ho Kim, Sang-Hun Lee

**Affiliations:** 1 Department of Emergency Medicine, Kyungpook National University Hospital, School of Medicine, Kyungpook National University, Daegu, Republic of Korea; 2 Department of Biostatistics, School of Medicine, Medical Research Collaboration Center, Kyungpook National University, Daegu, Republic of Korea; 3 Department of Public Health, Kyungpook National University Hospital, School of Medicine, Kyungpook National University, Daegu, Republic of Korea; 4 Department of Emergency Medicine, Kyungpook National University Chilgok Hospital, School of Medicine, Kyungpook National University, Daegu, Republic of Korea; 5 Department of Emergency Medicine, Yeungnam University College of Medicine, Daegu, Republic of Korea; 6 Department of Emergency Medicine, Keimyung University Dongsan Medical Center, Keimyung University School of Medicine, Daegu, Republic of Korea

**Keywords:** Out-of-hospital cardiac arrest: emergency medical services, Survival, Advanced cardiac life support

## Abstract

**Background:**

Prehospital factors play a vital role in out-of-hospital cardiac arrest (OHCA) survivability, and they vary between countries and regions. We investigated the prehospital factors associated with OHCA outcomes in a single metropolitan city in the Republic of Korea.

**Methods:**

This study included adult medical OHCA patients enrolled prospectively, using data from the citywide OHCA registry for patients registered between 2018 and 2021. The primary outcome was survival to hospital discharge. Multivariable logistic regression analysis was conducted to determine the factors associated with the study population’s clinical outcomes, adjusting for covariates. We performed a sensitivity analysis for clinical outcomes only for patients without prehospital return of spontaneous circulation prior to emergency medical service departure from the scene.

**Results:**

In multivariable logistic regression analysis, older age (odds ratio [OR] 0.96; 95% confidence interval [CI] 0.95–0.97), endotracheal intubation (adjusted odds ratio [aOR] 0.29; 95% [CIs] 0.17–0.51), supraglottic airway (aOR 0.29; 95% CI 0.17–0.51), prehospital mechanical chest compression device use (OR 0.13; 95% CI 0.08–0.18), and longer scene time interval (OR 0.96; 95% CI 0.93–1.00) were negatively associated with survival. Shockable rhythm (OR 24.54; 95% CI 12.99–42.00), pulseless electrical activity (OR 3.11; 95% CI 1.74–5.67), and witnessed cardiac arrest (OR 1.59; 95% CI 1.07–2.38) were positively associated with survival. In the sensitivity analysis, endotracheal intubation, supraglottic airway, prehospital mechanical chest compression device use, and longer scene time intervals were associated with significantly lower survival to hospital discharge.

**Conclusions:**

Regional resuscitation protocol should be revised based on the results of this study, and modifiable prehospital factors associated with lower survival of OHCA should be improved.

**Supplementary Information:**

The online version contains supplementary material available at 10.1186/s12873-023-00899-3.

## Introduction

Out-of-hospital cardiac arrest (OHCA) is one of the leading causes of death worldwide [1, 2]. Although the treatment of OHCA has increased significantly recently, the survival of OHCA remains at 10% even in developed countries [3, 4]. Many studies have been conducted to identify factors that may improve the chance of survival after OHCA. Age, witness status, bystander cardiopulmonary resuscitation (CPR), shockable initial rhythm, early defibrillation by automated external defibrillator (AED), and ambulance response time are reportedly associated with survival of OHCA [5–8]. While some factors have been confirmed as predictors of an increased chance of survival, the role of many remains unclear.

Survival after OHCA can be affected by prehospital and hospital factors. These are related to the patient (age, sex, ethnicity etc.), event (location of arrest, witness status, bystander CPR), system (quality of CPR, emergency medical service [EMS] system, dispatcher-assisted CPR), and to therapeutic factors (pharmacotherapy, airway management, quality of in-hospital care) [1]. The survival of OHCA has been shown to vary between countries or regions and is attributable to differences in the prehospital and hospital factors. The contribution of each factor to the outcomes of OHCA remains unclear [9–12]. The benefits of some factors are self-evident, such as the rate of bystander CPR, but some are non-intuitive, although they can potentially help. Several randomized control trials (RCT) have been conducted to determine the effects of prehospital drugs and procedures, such as prehospital epinephrine administration [13], advanced airway management (AAM) [14, 15], and mechanical chest compression devices (MCD) [16, 17]. Outcome might also be influenced by unmeasured factors such as the proficiency of EMS personnel. EMS system difference may also affect OHCA outcomes [18].

As prehospital factors play a vital role in OHCA survivability, this study aimed to determine the factors associated with OHCA outcomes in a single metropolitan city in the Republic of Korea, focusing on the prehospital and EMS phases.

## Methods

### Study design and setting

This was a retrospective, observational study using a citywide, prospective, population-based clinical registry. We enrolled patients who experienced OHCA between January 2018 and December 2021. In 2014, the Daegu Emergency Medicine Collaboration Committee launched the Daegu EMS Registry (DEMSRe), which was a prospective, citywide, population-based, volunteer-based clinical registry for patients with OHCA transported to the participating emergency department (ED) via EMS. Its data comes from the EMS run sheet, dispatcher CPR registry, and hospital medical records according to the Utstein guidelines for reporting of cardiac arrest. Daegu is located on the southern-eastern side of the Republic of Korea and encompasses 883.6 km^2^, which had a population of 2,410,700 in 2020. There are two regional emergency medical centers and four local emergency medical centers in the city, and all emergency centers participate in DEMSRe.

As of 2021, the Daegu metropolitan city fire department had 8 fire stations, 48 safety centers, and 47 ambulances. Each EMS team comprised three members, including a level-1 emergency medical technician (EMT) (equivalent to the United States advanced EMT) and a level-2 EMT (similar to an EMT). Each EMS team included at least one level-1 EMT. The level-1 EMT could perform advanced airway insertion and intravenous (IV) access under medical direction. After September 2019, level-1 EMTs were authorized to administer IV epinephrine under medical direction. A dual-dispatch system involved two ambulances being dispatched to the scene if available nearby when an OHCA emergency call was received by the dispatch center. Every EMS team had at least one MCD.

### Participants

All adult OHCA patients 18 years and older who enrolled in the registry were included in this study. We excluded patients whose resuscitation was not attempted by the EMS, patients whose cardiac arrest was witnessed by the EMS, patients with non-cardiac etiologies (e.g., trauma, asphyxia, poisoning), and patients without information of the results of hospital discharge.

### Data variables

We collected age, sex, initial electrocardiography (ECG) rhythm, witness status, location of cardiac arrest, bystander CPR status, type of bystander CPR, prehospital defibrillation, type of prehospital airway management, prehospital epinephrine use, and prehospital MCD use. We also obtained details on dual-dispatch status, response time interval (RTI), scene time interval (STI) and transport time interval (TTI), prehospital return of spontaneous circulation (ROSC), survival to hospital discharge, and good neurologic outcome at hospital discharge. RTI was defined as the time from emergency call to EMS arrival at the scene. STI was defined as the time from EMS arrival at the scene to EMS departure for the hospital. TTI was defined as the time from EMS departure to arrival at the ED.

### Outcomes

The primary outcome was survival to hospital discharge. The secondary outcome was a good neurologic outcome at hospital discharge, which was defined as Cerebral Performance Category scale 1 or 2.

### Statistical analysis

All statistical analyses were performed using the R software version 4.1.3 (R Foundation for Statistical Computing, Vienna, Austria). The demographics and baseline characteristics of the study population were presented using descriptive analysis. Categorical variables were presented as frequencies and percentages. Pearson’s chi-square test was used for the analysis. Continuous variables were presented as medians and interquartile ranges (IQR, 25th and 75th percentiles) with the Mann–Whitney U-test used for analysis according to the result of the Shapiro–Wilk test for normality. To determine the factors associated with the outcomes for patients with OHCA, we used multivariable logistic regression analyses and calculated adjusted odds ratios (aOR) and 95% confidence intervals (CIs) after adjusting for age, sex, initial ECG rhythm, witness, bystander CPR, location of cardiac arrest, prehospital IV epinephrine administration, type of prehospital airway management, prehospital mechanical CPR (MCPR), dual-dispatch, RTI, STI, and TTI. We performed sensitivity analysis for primary and secondary outcomes only for patients without prehospital ROSC prior to EMS departure from the scene, since the presence of early ROSC at the scene may affect the choice of the prehospital procedure or time interval.

### Ethical statement

This study was approved by the Institutional Review Board (IRB) of Kyungpook National University Hospital (IRB No. 2016-03-027) and performed in accordance with the provisions of the Declaration of Helsinki. The requirement for informed consent was waived by the IRB because of the retrospective, observational nature of the study.

## Results

**Fig. 1:**
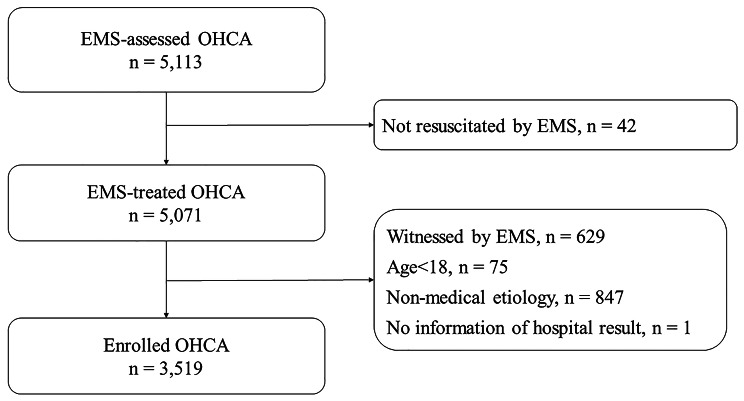
Study flow diagram. Abbreviations: EMS, emergency medical service; OHCA, out-of-hospital cardiac arrest

There were 5,113 EMS-assessed OHCA within the study period. We excluded the patients whose resuscitation was not attempted by EMS (n = 42), patients whose cardiac arrest was witnessed by the EMS (n = 629), patients who were < 19 years old (n = 75), patients with non-cardiac etiologies (n = 847), and patients without information about hospital discharge (n = 1). A total of 3,519 OHCA met the inclusion criteria and were enrolled in the final analysis (Fig. 1).

### Demographics and baseline characteristics of the study population

The demographics and baseline characteristics of the study population are shown in Table 1. There were 2,241 (63.7%) males and the median age was 74.0. Most of the cardiac arrests happened at home (2,572, 73.1%). The witnessed arrests were 48.0%, and the bystander CPR rate was 61.9%. In terms of EMS resuscitation, dual-dispatch was provided in most cases (96.1%). The EMS administered epinephrine in 51.0% of cases. Supraglottic airway (SGA) was the most common prehospital airway management, with 2,046 (58.1%) cases, followed by endotracheal intubation (ETI) with 1,235 (35.1%) and bag valve mask (BVM) with 238 (6.8%). Prehospital MCPR was performed in 2,759 patients (78.4%). The prehospital ROSC rate was 12.0%, survival to hospital discharge, 8.4%, and good neurologic outcome, 6.5%.

**Table 1:** Demographics and baseline characteristics of study population

	Study population (n = 3,519)
Year	
2018	883 (25.1)
2019	847 (24.1)
2020	910 (25.9)
2021	879 (25.0)
Sex, male	2,241 (63.7)
Age	74.0 (61.0–81.0)
Place	
Home	2,572 (73.1)
Public	947 (26.9)
Witness, yes	1,688 (48.0)
Initial ECG rhythm	
Shockable	575 (16.3)
PEA	779 (22.1)
Asystole	2,154 (61.2)
Unknown	11 (0.3)
Bystander CPR, yes	2,179 (61.9)
Dual-dispatch, yes	3,382 (96.1)
Prehospital defibrillation, yes	767 (21.8)
EMS epinephrine use, yes	1,794 (51.0)
Prehospital airway management	
BVM	238 (6.8)
SGA	2,046 (58.1)
ETI	1,235 (35.1)
Mechanical CPR, yes	2,759 (78.4)
RTI	8.0 (6.0–10.0)
STI	17.0 (14.0–20.0)
TTI	7.0 (4.0–10.0)
Clinical outcomes	
Prehospital ROSC	424 (12.0)
Survival to hospital discharge	297 (8.4)
Good neurologic outcome	228 (6.5)

Values are presented as frequency (%) or median (interquartile range). Abbreviations: ECG, electrocardiography; PEA, Pulseless electrical activity; CPR, cardiopulmonary resuscitation; EMS, emergency medical service; BVM, bag valve mask; SGA, supraglottic airway; ETI, endotracheal intubation; RTI, response time interval; STI, scene time interval; TTI, transport time interval; ROSC, recovery of spontaneous circulation

### Comparison of factors associated with clinical outcomes

Table 2 shows the results of the comparison of demographics and baseline characteristics between groups according to clinical outcomes. Male, witnessed arrest, public place, shockable rhythm, and bystander CPR showed significantly higher rates of survival to hospital discharge (*P* < 0.001). The survival to hospital discharge of the prehospital epinephrine group was 4.7%, which was lower than that of the no prehospital epinephrine group of 12.3% (*P* < 0.001). The survival to hospital discharge of the MCPR group was 2.3%, which was much lower than the manual CPR group of 30.8% (*P* < 0.001). In prehospital airway management, the survival to hospital rate in the BVM group was the highest at 44.1%, followed by ETI at 6.2% and SGA at 5.6% (*P* < 0.001). In all clinical outcomes, both RTI and STI were significantly shorter in the survival group than in the death group (*P* < 0.001).

**Table 2:** Comparison of factors of associated with OHCA clinical outcomes

	Prehospital ROSC			Survival to hospital discharge			Good neurological outcome		
	Yes (n = 424)	No (n = 3,095)	P	Yes (n = 297)	No (n = 3,222)	P	Yes (n = 228)	No (n = 3,291)	P
Sex			0.001			< 0.001			< 0.001
Male	300 (13.4)	1,941 (86.6)		236 (10.5)	2005 (89.5)		188 (8.4)	2,053 (91.6)	
Female	124 (9.7)	1,154 (90.3)		61 (4.8)	1217 (95.2)		40 (3.1)	1,238 (96.9)	
Age	62.0 (52.0–73.5)	75.0 (63.0–82.0)	< 0.001	58.0 (50.0–67.0)	75.0 (63.0–82.0)	< 0.001	56.0 (49.0–65.0)	75.0 (63.0–82.0)	< 0.001
Place			< 0.001			< 0.001			< 0.001
Home	245 (9.5)	2,327 (90.5)		156 (6.1)	2,416 (93.9)		113 (4.4)	2,459 (95.6)	
Public	179 (18.9)	768 (81.1)		141 (14.9)	806 (85.1)		115 (12.1)	832 (87.9)	
Witness			< 0.001			< 0.001			< 0.001
Yes	305 (18.1)	1,383 (81.9)		237 (14.0)	1,451 (86.0)		195 (11.6)	1,493 (88.4)	
No	119 (6.5)	1,712 (93.5)		60 (3.3)	1,771 (96.7)		33 (1.8)	1,798 (98.2)	
Initial ECG rhythm^*^			< 0.001			< 0.001			< 0.001
Shockable	243 (42.3)	332 (57.7)		238 (41.4)	337 (58.6)		204 (35.5)	371 (64.5)	
PEA	85 (10.9)	694 (89.1)		36 (4.6)	743 (95.4)		19 (2.4)	760 (97.6)	
Asystole	92 (4.3)	2,062 (95.7)		21 (1.0)	2,133 (99.0)		3 (0.1)	2,151 (99.9)	
Bystander CPR			< 0.001			< 0.001			< 0.001
Yes	318 (14.6)	1,861 (85.4)		236 (10.8)	1,943 (89.2)		185 (8.5)	1,994 (91.5)	
No	106 (7.9)	1,234 (92.1)		61 (4.6)	1,279 (95.4)		43 (3.2)	1,297 (96.8)	
Dual-dispatch			0.790			0.217			0.200
Yes	406 (12.0)	2,976 (88.0)		281 (8.3)	3,101 (91.7)		215 (6.4)	3,167 (93.6)	
No	18 (13.1)	119 (86.9)		16 (11.7)	121 (88.3)		13 (9.5)	124 (90.5)	
Prehospital defibrillation			< 0.001			< 0.001			< 0.001
Yes	258 (33.6)	509 (66.4)		244 (31.8)	523 (68.2)		206 (26.9)	561 (73.1)	
No	166 (6.0)	2,586 (94.0)		53 (1.9)	2,699 (98.1)		22 (0.8)	2,730 (99.2)	
EMS Epinephrine use			0.011			< 0.001			< 0.001
Yes	191 (10.6)	1,603 (89.4)		84 (4.7)	1,710 (95.3)		50 (2.8)	1,744 (97.2)	
No	233 (13.5)	1,492 (86.5)		213 (12.3)	1,512 (87.7)		178 (10.3)	1,547 (89.7)	
Prehospital airway management			< 0.001			< 0.001			< 0.001
BVM	114 (47.9)	124 (52.1)		105 (44.1)	133 (55.9)		100 (42.0)	138 (58.0)	
SGA	159 (7.8)	1,887 (92.2)		115 (5.6)	1,931 (94.4)		75 (3.7)	1,971 (96.3)	
ETI	151 (12.2)	1,084 (87.8)		77 (6.2)	1,158 (93.8)		53 (4.3)	1,182 (95.7)	
Mechanical CPR			< 0.001			< 0.001			< 0.001
Yes	122 (4.4)	2,637 (95.6)		63 (2.3)	2,696 (97.7)		30 (1.1)	2,729 (98.9)	
No	302 (39.7)	458 (60.3)		234 (30.8)	526 (69.2)		198 (26.1)	562 (73.9)	
RTI	7.5 (6.0– 9.0)	8.0 (6.0–10.0)	< 0.001	8.0(6.0–9.0)	8.0(6.0–10.0)	< 0.001	7.0 (6.0–9.0)	8.0 (6.0–10.0)	< 0.001
STI	15.0 (12.0–19.0)	17.0 (14.0–20.0)	< 0.001	14.0(11.0–18.0)	17.0(14.0–20.0)	< 0.001	14.0 (10.0–18.0)	17.0 (14.0–20.0)	< 0.001
TTI	7.0 (5.0–11.0)	7.0 (4.0–10.0)	0.009	7.0(4.0–11.0)	7.0(4.0–10.0)	0.358	7.0 (4.0–11.0)	7.0 (4.0–10.0)	0.253

^*^The missing data were 11(0.3%). Values are presented as frequency (%) or median (interquartile range). Abbreviations: ROSC, recovery of spontaneous circulation; ECG, electrocardiography; PEA, pulseless electrical activity; CPR, cardiopulmonary resuscitation; EMS, emergency medical service; BVM, bag valve mask; SGA, supraglottic airway; ETI, endotracheal intubation; RTI, response time interval; STI, scene time interval; TTI, transport time interval

### Multivariable logistic regression analysis of factors associated with clinical outcomes

Table 3 shows the multivariable logistic regression analysis of the factors associated with OHCA clinical outcomes. Older age (OR 0.96; 95% CI 0.95–0.97) was negatively associated with survival, but shockable rhythm (OR 24.54; 95% CI 12.99–42.00), pulseless electrical activity (PEA) (OR 3.11; 95% CI 1.74–5.67), and witnessed cardiac arrest (OR 1.59; 95% CI 1.07–2.38) were positively associated with survival. Regarding prehospital advanced procedures, survival to hospital discharge was significantly lower for ETI (OR 0.29; 95% CI 0.17–0.51), SGA (OR 0.29; 95% CI 0.17–0.51) than BVM, and MCPR (OR 0.13; 95% CI 0.08–0.18) was also associated with decreased survival to hospital discharge. A longer STI was associated with worse survival to hospital discharge (OR 0.96; 95% CI 0.93–1.00). Prehospital epinephrine administration was associated with significantly improved prehospital ROSC (OR 2.78; 95% CI 2.03–3.84), but not significantly associated with survival to hospital charge and good neurological outcomes.

**Table 3:** Multivariable logistic regression analysis of OHCA clinical outcomes

	Prehospital ROSC	Survival to hospital discharge	Good neurological outcome
	aOR (95% CI)	aOR (95% CI)	aOR (95% CI)
Sex			
Female	1.00	1.00	
Male	0.95 (0.71–1.27)	1.48 (0.98–2.27)	2.02 (1.17–3.55)
Age	0.98 (0.97–0.99)	0.96 (0.95–0.97)	0.95 (0.94–0.97)
Initial ECG			
Asystole	1.00	1.00	
Shockable	6.46 (4.64–9.04)	24.54 (12.99–42.00)	114.16 (40.96–477.02)
PEA	1.88 (1.33–2.66)	3.11 (1.74–5.67)	9.51 (3.04–42.00)
Witness, Yes	1.51 (1.13–2.01)	1.59 (1.07–2.38)	2.34 (1.37–4.07)
Place			
Home	1.00	1.00	1.00
Public	1.31 (0.99–1.72)	1.37 (0.96–1.97)	1.48 (0.95–2.31)
Bystander CPR, Yes	1.31 (0.98–1.76)	1.35 (0.90–2.04)	1.18 (0.70–2.00)
Prehospital airway management			
BVM	1.00	1.00	1.00
ETI	0.40 (0.25–0.62)	0.29 (0.17–0.51)	0.19 (0.10–0.38)
SGA	0.25 (0.16–0.39)	0.29 (0.17–0.51)	0.17 (0.09–0.31)
Prehospital epinephrine, Yes	2.78 (2.03–3.84)	0.87 (0.58–1.30)	0.66 (0.40–1.10)
Prehospital MCD, Yes	0.09 (0.07–0.12)	0.13 (0.08–0.18)	0.09 (0.06–0.16)
Dual-dispatch, Yes	2.77 (1.46–5.45)	2.41 (1.14–5.31)	2.50 (1.03–6.37)
RTI	0.96 (0.91–1.00)	0.95 (0.89–1.01)	0.96 (0.88–1.04)
STI	0.98 (0.95–1.00)	0.96 (0.93–1.00)	0.97 (0.93–1.01)
TTI	1.00 (1.00–1.00)	1.00 (1.00–1.00)	1.00 (1.00–1.01)

Abbreviations: ROSC, recovery of spontaneous circulation; aOR, adjusted odds ratio; CI, confidence interval; ECG, electrocardiography; PEA, pulseless electrical rhythm; CPR, cardiopulmonary resuscitation; BVM, bag valve mask; ETI, endotracheal intubation; SGA, supraglottic airway; MCD, mechanical chest compression device; RTI, response time interval; STI, scene time interval; TTI, transport time interval

### Sensitivity analysis (only done for the patients without ROSC at the scene)

In the sensitivity analysis for OHCA patients without ROSC before departure from the scene, ETI (OR 0.22; 95% CI 0.09–0.50) and SGA (OR 0.20; 95% CI 0.09–0.45) use still showed significantly lower survival to hospital discharge and good neurological outcomes than BVM. The prehospital MCPR (OR 0.36; 95% CI 0.21–0.63) was also associated with significantly lower survival to hospital discharge. A longer STI was associated with a significantly lower survival to hospital discharge (OR 0.94; 95% CI 0.90–1.00) (Table 4).

**Table 4:** Sensitivity analysis (including only the patients without ROSC at the scene)

	Survival to hospital discharge	Good neurological outcome
	aOR (95% CI)	aOR (95% CI)
Bystander CPR, Yes	1.35 (0.77–2.39)	1.22 (0.49–3.05)
Prehospital airway management		
BVM	1.00	1.00
ETI	0.22 (0.09–0.50)	0.07 (0.02–0.26)
SGA	0.20 (0.09–0.45)	0.04 (0.01–0.16)
Prehospital epinephrine, Yes	1.08 (0.62–1.86)	0.84 (0.36–1.97)
Prehospital MCD use, Yes	0.36 (0.21–0.63)	0.19 (0.08–0.44)
Dual-dispatch, Yes	2.39 (0.70–8.16)	3.47 (0.55–21.94)
RTI	0.93 (0.85–1.02)	0.96 (0.83–1.09)
STI	0.94 (0.90–1.00)	0.98 (0.91–1.05)
TTI	1.00 (0.99–1.01)	1.00 (0.99–1.02)

Abbreviations: ROSC, recovery of spontaneous circulation; aOR, adjusted odds ratio; CI, confidence interval; CPR, cardiopulmonary resuscitation; BVM, bag valve mask; ETI, endotracheal intubation; SGA, supraglottic airway; MCD, mechanical chest compression device; RTI, response time interval; STI, scene time interval; TTI, transport time interval

## Discussion

We determined the prehospital factors associated with survival in OHCA using a population-based registry of a single metropolitan city. We found that the significant factors associated with higher survival were younger age, shockable rhythm and PEA on the initial ECG rhythm, witnessed cardiac arrest, and dual dispatch. The significant factors associated with lower survival were ETI and SGA in prehospital AAM, prehospital MCPR, and longer STI. Prehospital MCPR and AAM, including ETI and SGA, were significantly negatively associated with good neurological outcomes and prehospital ROSC, as well as survival to hospital discharge.

In this study, the rate of AAM was very high at 93.2%, which was much higher than reported in previous studies [19, 20]. In the Pan-Asian Resuscitation Outcomes Study, the rate of prehospital AAM in seven Asian countries varied appreciably [18]; the highest rate of prehospital AAM was 82.5% in Singapore, which was lower than in our result. In a study using national data in the Republic of Korea, the prehospital AAM was 65.0%. Our city’s rate was higher than that of other countries and the domestic average [21]. However, it is not clear whether better prehospital AAM leads to better outcomes in OHCA. In Osaka (65.0%) and Singapore (84.9%), where prehospital AAM was higher, prehospital AAM was negatively associated with neurological recovery of OHCA; however, the association between prehospital AAM and neurological recovery of OHCA was not significant in Seoul (19.2%) and Taipei (34.1%), which had a relatively lower prehospital AAM [22]. These results suggest that a high frequency of AAM does not necessarily guarantee good outcomes. On the other hand, Onoe et al. reported that the prefectures with high frequency of prehospital AAM were associated with neurologically favorable survival for OHCA in Japan [19]. In some previous observational studies, AAM did not show significant differences in OHCA outcomes compared with non-AAM, or in some cases were associated with worse outcomes [23–25]. Although the reason for the regional variability in the effect of prehospital AAM is not apparent, it has been reported that the type and process of AAM, and the proficiency and teamwork of EMS providers may contribute to the differences in results [22]. We have found that AAM was associated with worse outcomes regardless of the type. If ROSC was achieved early, prehospital AAM may not have been attempted, and better outcomes may have been reported with non-AAM. Therefore, we performed additional sensitivity analysis only for OHCA without ROSC before departure from the field. It confirmed that AAM had a negative effect on OHCA even after adjusting for identifiable factors. However, the success rate and number of attempts for AAM, especially ETI, could not be determined using our registry. These factors might introduce significant bias for the negative results obtained for prehospital AAM in this study. Behrens et al. reported that prehospital ETI was associated with a higher survival rate than laryngeal (LT) placement in OHCA based on the German Resuscitation Registry (GRR) [26]. Notably, most countries participating in the GRR have physician-based EMS system, which was reported to have a 99% success rate for ETI within two attempts in another study on the GRR [27]. Conversely, the paramedic-based Prehospital Airway Resuscitation Trial (PART) demonstrated that LT placement increased the survival rate (18.3% vs. 15.4%) and generated favorable neurologic outcomes (7.1% vs. 5.0%) compared with ETI [14]. However, PART reported the success rate of ETI as merely 51%. These findings suggest that different Ems system (physician-based vs. paramedic-based) and the proficiency level of EMS member may contribute to the variation in the effectiveness of prehospital AAM.

Prehospital MCPR was also associated with worse outcomes. Previous studies found that CPR with MCD did not improve clinical outcomes compared to manual CPR [17, 28]. The American Heart Association (AHA) guideline recommends that MCD may be considered in specific settings where the delivery of high-quality manual compression may be challenging or dangerous for the provider [29]. During the COVID-19 pandemic, the AHA recommended replacing manual devices with MCD to reduce the number of rescuers performing resuscitation. In our city, during the COVID-19 pandemic, MCD use was actively recommended under agreement with medical institutions and EMS. Moreover, we found that the frequency of MCD use increased after the COVID-19 pandemic [30]. In this study, we performed additional analyzes to determine the impact of the COVID-19 pandemic on each factor and found prehospital MCPR was still a factor associated with worse survival regardless of the COVID-19 outbreak (additional file 1). There are two plausible reasons for this. Firstly, major factors that negatively affect MCD use include interruption and delay of chest compressions for device deployment [28]. Huang et al. reported that it took an average of 122.6 s to deploy the MCD and more than 70% of them had no-flow time [31]. The significant time wasted on MCD deployment led to a long no-flow time. This suggests that training on MCD placement and positioning is necessary to improve the effectiveness of MCD. Secondly, malposition of the device may cause poor chest compression and counteract the potential benefits of MCPR. Blomberg et al. reported that LUCAS-CPR had a higher fraction of inappropriate chest compressions than manual CPR in the manikin simulation study [32]. If the EMS providers failed to correct the MCD position or the MCD was repositioned during transport, a long period of poor chest compressions could adversely affect outcomes.

In a large, multicenter, double-blinded RCT comparing epinephrine with placebo, the use of prehospital epinephrine was associated with better survival than the use of placebo despite an insignificant effect on favorable neurologic outcome [13]. Several studies have reported that early epinephrine administration was associated with increased neurologically favorable survival in non-shockable OHCA or all OHCA [33, 34]. The current guidelines recommend rapid epinephrine administration in adult patients with non-shockable OHCA [29]. However, prehospital epinephrine administration was associated with a higher prehospital ROSC, but did not resulted in the significant improvement in the rate of survival to hospital discharge and good neurologic outcome in this study. Our finding is consistent with previous several observational studies. Hagihara et al. reported that use of prehospital epinephrine was associated with increased chance of prehospital ROSC, but decreased survival and good neurologic outcomes in Japan [35]. Han et al. also reported that prehospital epinephrine administration was associated with decreased survival in OHCA in the Republic of Korea; the authors suggested that the difference from previous studies demonstrating the effectiveness of prehospital epinephrine was due to differences in the EMS systems and performance of EMS providers [36]. Recently, Knapp et al. developed a statistical methodology to compare survival rates of OHCA patients treated using different EMS systems [37]. They indicated that the survival rate of 3.2% and favorable neurologic outcome of 2.2% reported in the epinephrine group in previous RCT could have been improved using another EMS system. This attempt will help in understanding the discrepancies between populations and facilitate the comparison of clinical outcomes between countries or regions worldwide in the future.

The poor outcome of prehospital epinephrine administration was likely to be associated with a longer STI. In a study by de Graaf et al., the authors suggested STI should be 8 to 15 min because most survivors achieved prehospital ROSC within the first 15 min of EMS resuscitation [38]. Jang et al. reported that on-scene ALS of more than 19 min was associated with worse neurologic outcomes [39]. Coute et al. reported that an STI > 20 min was likely to be associated with worse neurologic outcomes in bystander-witnessed OHCA [40]. Although there are slight differences depending on the study population and country, a longer STI was likely to be associated with worse OHCA patient survival. In this study, STI was 3 min longer in the surviving group than in the death group (17 min vs. 14 min). We found that a 1 min increase in STI reduced the odds of survival by 3.9% (aOR 0.961; 95% CI 0.927 − 0.995) in the adjusted model, and a similar pattern was observed in the sensitivity analysis of excluded patients who achieved ROSC at the scene. The optimal STI for patients with OHCA is not clear, but STI during prehospital resuscitation efforts should not be unnecessarily prolonged.

Finally, we found the dual-dispatch was associated with increased survival in OHCA. Dual-dispatch aimed to reduce the response time and provide high-quality CPR at the scene [41]. A rapid EMS response time contributed to increased survival for OHCA patients [42, 43], but the association between dual-dispatch and high-quality CPR is unclear. Kim et al. reported that dual-dispatch was associated with favorable neurologic outcomes in OHCA when the on-scene time was less than 10 min [41]. Although the larger number of EMS providers enabled more advanced prehospital procedures to be performed, their effects were also unclear. In our study, dual-dispatch was provided for most OHCA patients, providing a setting in which more advanced prehospital procedures could be performed. However, the effect of prehospital epinephrine administration was insignificant, and AAM and use of MCDs were negative factors for surviving OHCA. As mentioned above, this may be a result of the difference in the EMS system and performance of EMS providers in advanced life support procedures. EMS providers should be trained in advanced skills and be deployed according to their proficiency. In addition, specific field resuscitation guidelines should be updated to set the number of times a procedure is performed and the time in which the goals should be achieved.

This study had some limitations. First, there might have been selection bias due to the retrospective nature of the study, despite adjustment for multiple potential confounders. Second, the results of this study cannot be generalized due to the difference in the performance of EMS providers and EMS system by country or region. Third, the number of attempts, success rate, and complication rate of prehospital AAM by EMS providers and the delay in providing chest compression when deploying MCD can significantly affect the outcome of cardiac arrest; however, this could not be evaluated with our data set.

## Conclusions

Our findings indicate that prehospital AAM, including ETI and SGA, use of prehospital MCD, and longer STI were negatively associated with survival to hospital discharge in our city. Based on these results, it is evident that modifications in the regional resuscitation protocol are warranted. However, these results should not be generalized to other countries owing to the difference in EMS system, and absence of EMS team’s performance assessment in this study. Future studies should determine the performance of prehospital advanced life support given by EMS providers and effect of prehospital factors by comparing different EMS system.

### Electronic supplementary material

Below is the link to the electronic supplementary material.

Supplementary Material 1

## Data Availability

The datasets which were analyzed during the current study are available from the corresponding author (Hyun Wook Ryoo, ryoo@knu.ac.kr) on reasonable request.

## Abbreviations

OHCA: Out-of-hospital cardiac arrest
CPR: Cardiopulmonary resuscitation
AED: Automated external defibrillator
EMS: Emergency medical service
RCT: Randomized control trial
AAM: Advanced airway management
MCD: Mechanical chest compression device
DEMSRe: Daegu EMS Registry
ED: Emergency department
EMT: Emergency medical technician
IV: Intravenous
ECG: Electrocardiography
RTI: Response time interval
STI: Scene time interval
TTI: Transport time interval
ROSC: Return of spontaneous circulation
MCPR: Mechanical CPR
SGA: Supraglottic airway
ETI: Endotracheal intubation
BVM: Bag valve mask
PEA: Pulseless electrical activity
