# Supplementary material for: Prehospital factors associated with out-of-hospital cardiac arrest outcomes in a metropolitan city: a 4-year multicenter study
Source: BMC Emerg Med. 2023 Oct 26;23:125. doi: 10.1186/s12873-023-00899-3 (PMC10601319; doi:10.1186/s12873-023-00899-3)
Supplement: Supplementary file 1 — Supplementary Material 1 [file 12873_2023_899_MOESM1_ESM.docx]

|  | Pre-COVID-19 | | COVID-19 period | |
| --- | --- | --- | --- | --- |
|  | Survival to hospital discharge | Good neurological outcome | Survival to hospital discharge | Good neurological outcome |
|  | aOR (95% CI) | aOR (95% CI) | aOR (95% CI) | aOR (95% CI) |
| Sex |  |  |  |  |
| Female | 1.00 | 1.00 | 1.00 | 1.00 |
| Male | 2.01 (1.10–3.79) | 2.14 (0.98–4.70) | 1.19 (0.65–2.21) | 2.22 (0.99–5.20) |
| Age | 0.96 (0.94–0.97) | 0.95 (0.93–0.97) | 0.96 (0.94–0.97) | 0.95 (0.93–0.97) |
| Initial ECG rhythm |  |  |  |  |
| Asystole | 1.00 | 1.00 | 1.00 | 1.00 |
| Shockable | 24.78 (12.44–53.69) | 248.01 (49.08–4577.77) | 23.14 (11.29–51.92) | 50.84 (14.59-322.45) |
| PEA | 2.40 (1.02–5.78) | 16.06 (2.69–310.89) | 4.05 (1.78-9.68) | 6.82 (1.61-46.93) |
| Witness, Yes | 2.20 (1.23–4.04) | 3.20 (1.48–7.29) | 1.17 (0.67-2.08) | 1.69 (0.79–3.78) |
| Place |  |  |  |  |
| Home | 1.00 | 1.00 | 1.00 | 1.00 |
| Public | 1.12 (0.64–1.91) | 1.40 (0.73–2.67) | 1.57 (0.94–2.61) | 1.52 (0.80–2.89) |
| Bystander CPR, Yes | 0.93 (0.51–1.69) | 1.09 (0.50–2.40) | 2.09 (1.16–3.85) | 1.42 (0.67–3.08) |
| Prehospital advanced airway |  |  |  |  |
| BVM | 1.00 | 1.00 | 1.00 | 1.00 |
| ETT | 0.35 (0.16–0.77) | 0.21 (0.08–0.54) | 0.41 (0.16–1.04) | 0.21 (0.06–0.62) |
| SGA | 0.24 (0.11–0.53) | 0.11 (0.04–0.29) | 0.37 (0.17–0.82) | 0.25 (0.10–0.60) |
| Prehospital epinephrine, Yes | 0.87 (0.46–1.60) | 0.80 (0.37–1.71) | 0.57 (0.32–1.02) | 0.38 (0.16–0.90) |
| Prehospital MCD use, Yes | 0.10 (0.06–0.18) | 0.08 (0.04–0.17) | 0.13 (0.07–0.23) | 0.10 (0.05–0.20) |
| Dual-dispatch, Yes | 2.03 (0.78–5.67) | 2.07 (0.66–7.27) | 4.57 (1.25–18.39) | 4.05 (0.96–18.63) |
| Response time interval | 0.95 (0.86–1.04) | 0.97 (0.86–1.09) | 0.92 (0.83–1.01) | 0.94 (0.82–1.06) |
| Scene time interval | 0.95 (0.88–1.01) | 0.95 (0.88–1.01) | 0.94 (0.89–0.99) | 0.97 (0.91–1.03) |
| Transport time interval | 1.00 (1.00–1.01) | 1.00 (1.00–1.01) | 0.99 (0.95–1.03) | 1.00 (0.95–1.01) |

**Table S1.** Subgroup analysis of OHCA outcomes according to the COVID-19 outbreak

aOR: adjusted odds ratio, CI: confidence interval, ECG: electrocardiography, PEA: pulseless electrical activity, CPR: cardiopulmonary resuscitation, BVM: bag valve mask, ETI: endotracheal intubation, SGA: supraglottic airway, MCD: Mechanical chest compression device
